# Use of patient-specific guides and 3D model in scapula osteotomy for symptomatic malunion

**DOI:** 10.1186/s41205-023-00184-w

**Published:** 2023-09-06

**Authors:** Stefano Cattaneo, Marco Domenicucci, Claudio Galante, Elena Biancardi, Alessandro Casiraghi, Giuseppe Milano

**Affiliations:** 1 Department of Bone and Joint Surgery, ASST Spedali Civili, Piazzale Spedali Civili 1, Brescia (BS), 25123 Italy; 2 Department of Medical and Surgical Specialties, Radiological Sciences, and Public Health, University of Brescia, Viale Europa 11, Brescia, 25123 BS Italy

**Keywords:** Scapula, Malunion, 3D model, Patient-specific guides

## Abstract

**Background:**

Scapular osteotomy for malunion can lead to resolution of pain and functional improvement in scapula fracture sequelae. Understanding three-dimensional bone morphology and analysing post-traumatic deformity is the main step of planning and the key to success of the procedure. 3D models and patient-specific guides are a growing technology to enhance accuracy of planning and to assist during surgery.

**Case presentation:**

We report the case of a 50 years old male, complaining of pain and limited function after a malunited scapular body fracture. Clinical assessment showed a severe impairment of shoulder function with active and passive forward flexion limited to 80°, absent external rotation, and internal rotation limited to the buttock. X-rays and CT scan showed an excessive lateral border offset of 53 mm and complete displacement of the glenoid segment anteriorly and medially to the scapular body, with impingement between the lateral most prominent scapular bone spur and humeral shaft. Glenopolar angle was 19°, scapular body angulation on the sagittal plane was 12°. Corrective osteotomy was planned on a virtual interactive rendering and on 3D printed models. Patient-specific guides were developed to perform a body-spine osteotomy with removal of a bone wedge, and a glenoid-spine osteotomy; a patient-specific wedge spacer was used to hold the reduction during plate fixation. Follow-up up to 12 months after surgery demonstrated improvement in scapula anatomy, shoulder girdle function, and patient-reported outcomes.

**Conclusions:**

For the first time in scapula malunion surgery, patient-specific osteotomy guides were succesfully used during surgery to perform osteotomies and to assist in reduction maneuvers.

## Background

Scapular fractures represent 0,5% of fractures [1] and are often the result of high energy trauma and multi combined injury [2]. Fractures of the scapular body or glenoid neck account from 62 to 98% of all scapula fractures [3–5], making extra-articular pattern of injury the most common one [6].

Although conservative treatment is a valuable option for a number of patients, quantitative measures for operative indication have been defined by several authors [7–12]. Failure to consider surgery when one or more criteria are present leads to malunion, functional and morphological impairment [13]. Osteotomy for symptomatic malunion of the scapula has been proposed by various authors in case reports and case series, and resulted in deformity correction, resolution of pain, improvement in function and in quality of life [13–16].

Preoperative planning is a crucial step, notably in segments with a complex three-dimensional morphology, as the scapula. The use of 3D printed model for the planning of scapula malunion surgery has been recently reported [19]. Performing osteotomies and assisting the procedure with patient-specific guides is an evolution of 3D modelling, described in several skeletal segments [17–21]. However, patient-specific guides have not been used in scapula osteotomy yet.

We report on the use of 3D planning and patient-specific guides to perform a scapular body osteotomy for post-traumatic malunion.

## Case presentation

**Fig. 1:**
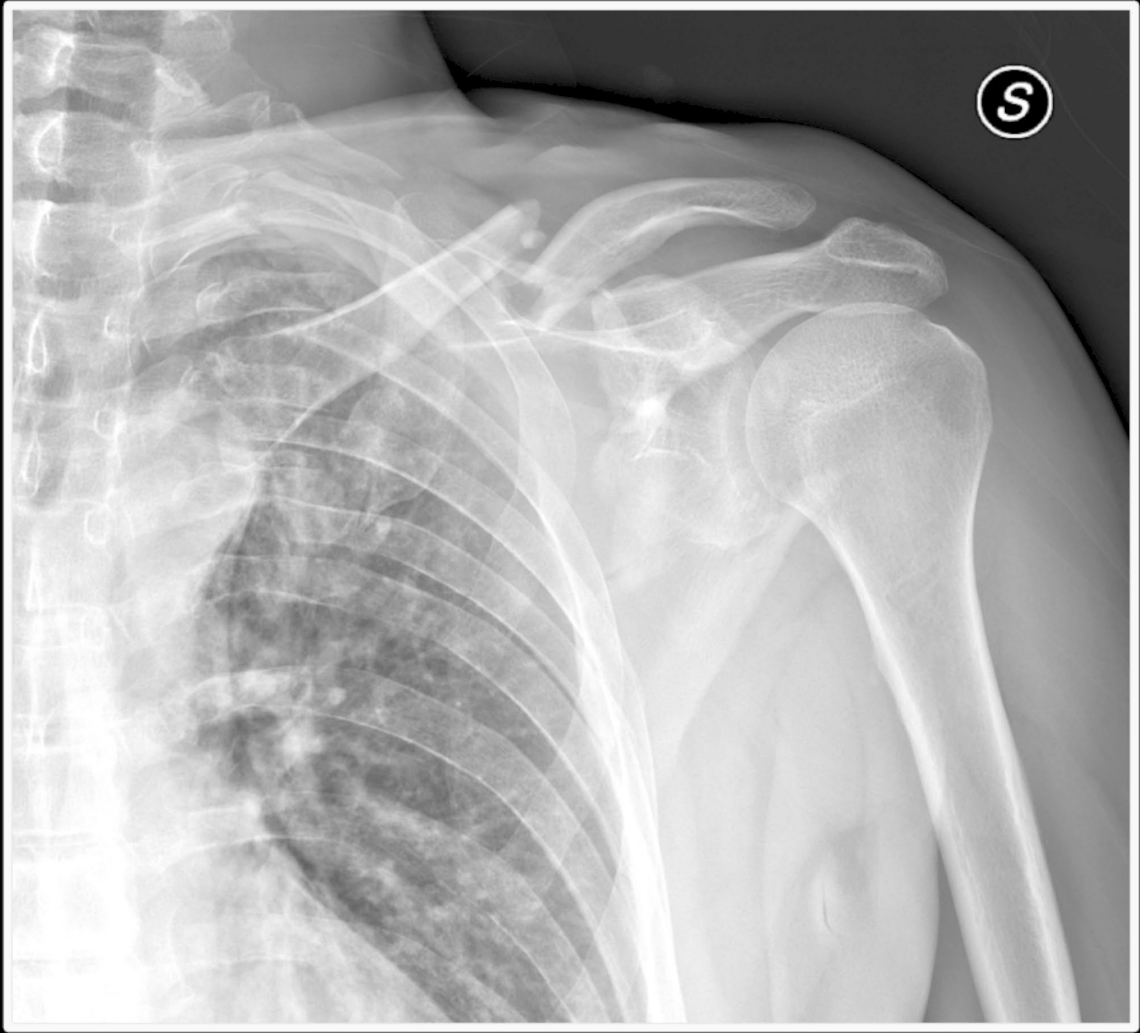
Initial X-ray of the left shoulder in AP view

A 50-year-old male was admitted to our department because of pain and limited range of movement of his left nondominant shoulder. Five months before he had been involved in a motor vehicle accident and underwent multiple lesions including moderate thoracic injury, occipital condyle fracture and left ipsilateral midshaft clavicle and scapular neck/body fracture (Fig. 1).

Shoulder girdle treatment, at the time of the accident, was limited to clavicle open reduction and internal fixation with plate and screws. Attempts at physical therapy were made, but were unsuccessful due to persistent stiffness at pain during mobilization.

Clinical assessment showed a severe impairment of shoulder function with active and passive forward flexion limited to 80° (normal 180°) [22], absent external rotation (normal 90°) [22], and internal rotation limited to the buttock.

The preoperative Disabilities of the Arm, Shoulder and Hand (DASH) Score was 73.3, whereas the Total Shoulder Pain and Disability Index (SPADI) was 88.5% (Total Pain Score 80%, Total Disability Score 93.8%). DASH Score and SPADI are self-reported questionnaires commonly used to measure upper limb and shoulder pain and disability; or both the scales the more positive is the outcome, the score decreases.

**Fig. 2:**
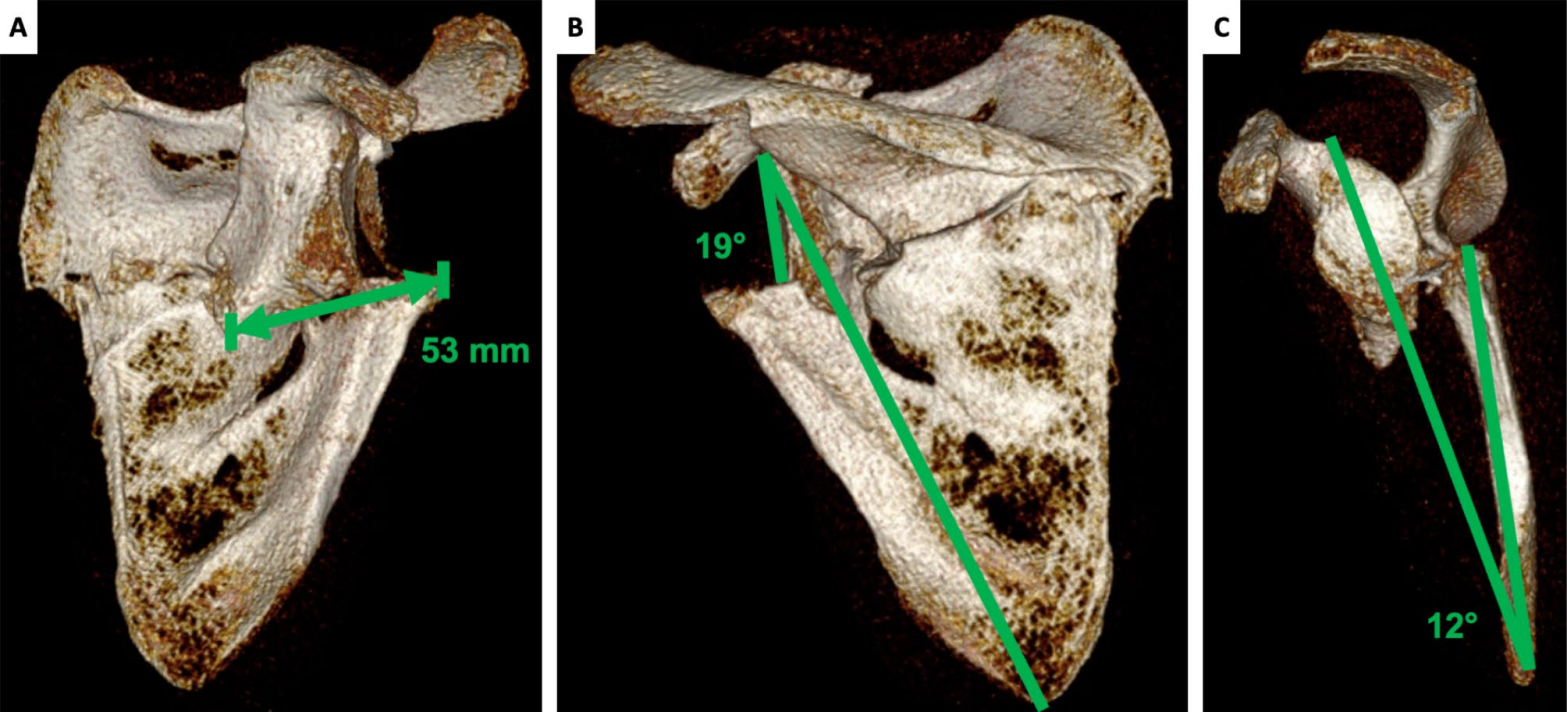
Left scapula deformity values measured on preoperative CT scan. A: lateral border offset 53 mm. B: glenopolar angle 19°. C: angulation deformity 12°

Radiographs and computed tomography (CT) scan showed a discrepancy between normal metrics for scapular imaging [13] and patient’s morphology with an excessive lateral border offset of 53 mm (cut off 20 mm) and complete displacement of the glenoid segment anteriorly and medially to the scapular body, with impingement between the lateral most prominent scapular bone spur and humeral shaft. Glenopolar angle was 19° (accepted when > 22°), scapular body angulation on the sagittal plane was 12° (should not exeed 45°) (Fig. 2).

**Fig. 3:**
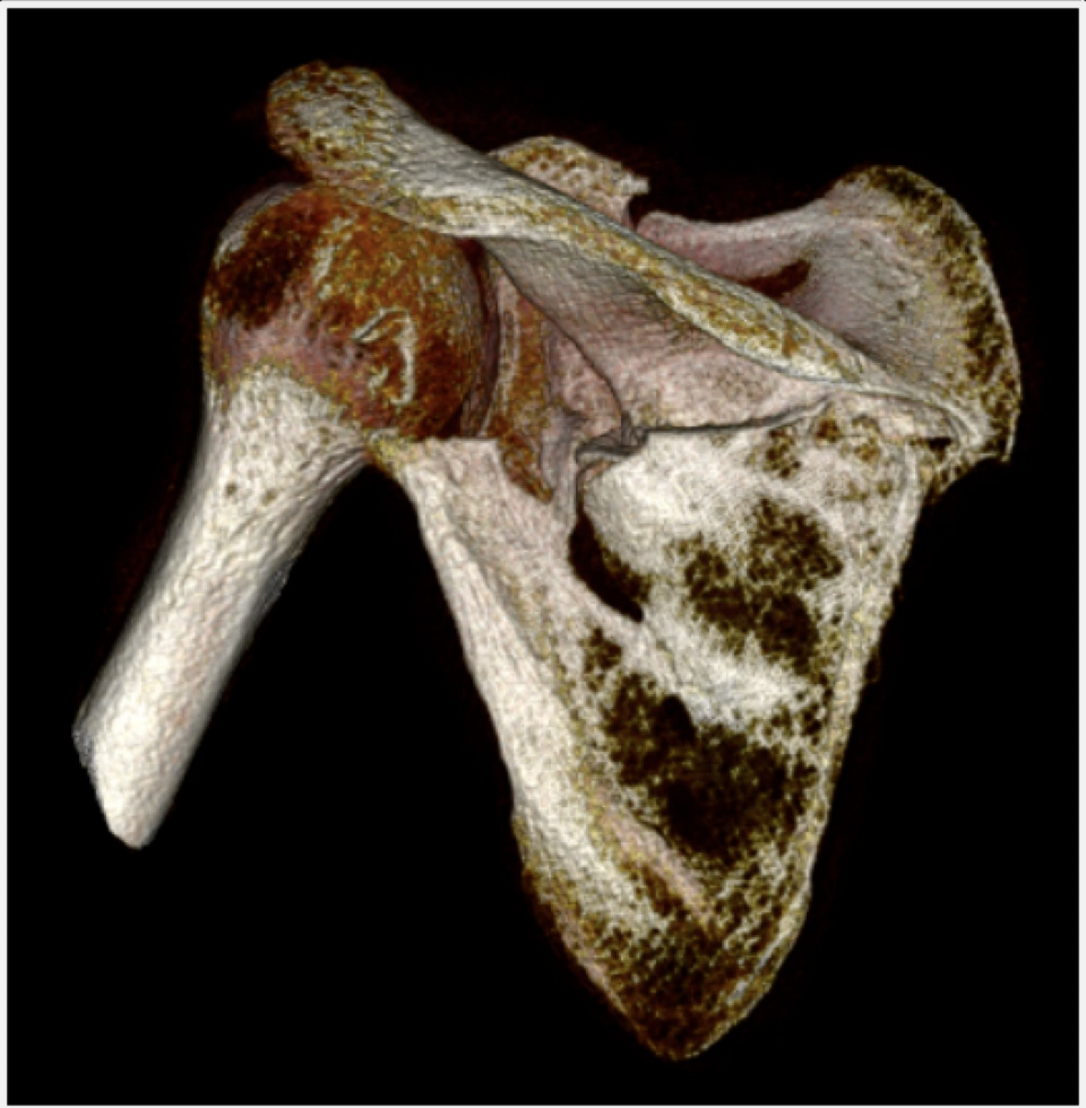
preoperative CT scan of the left scapula and humerus showing impingement between the lateral most prominent spur of the scapular body and the humerus

According to close to normal scapular body angulation and lower end of abnormal glenopolar angle, pain, disability and surgical indication seemed to arise from scapulo-humeral impingement (Fig. 3).

**Fig. 4:**
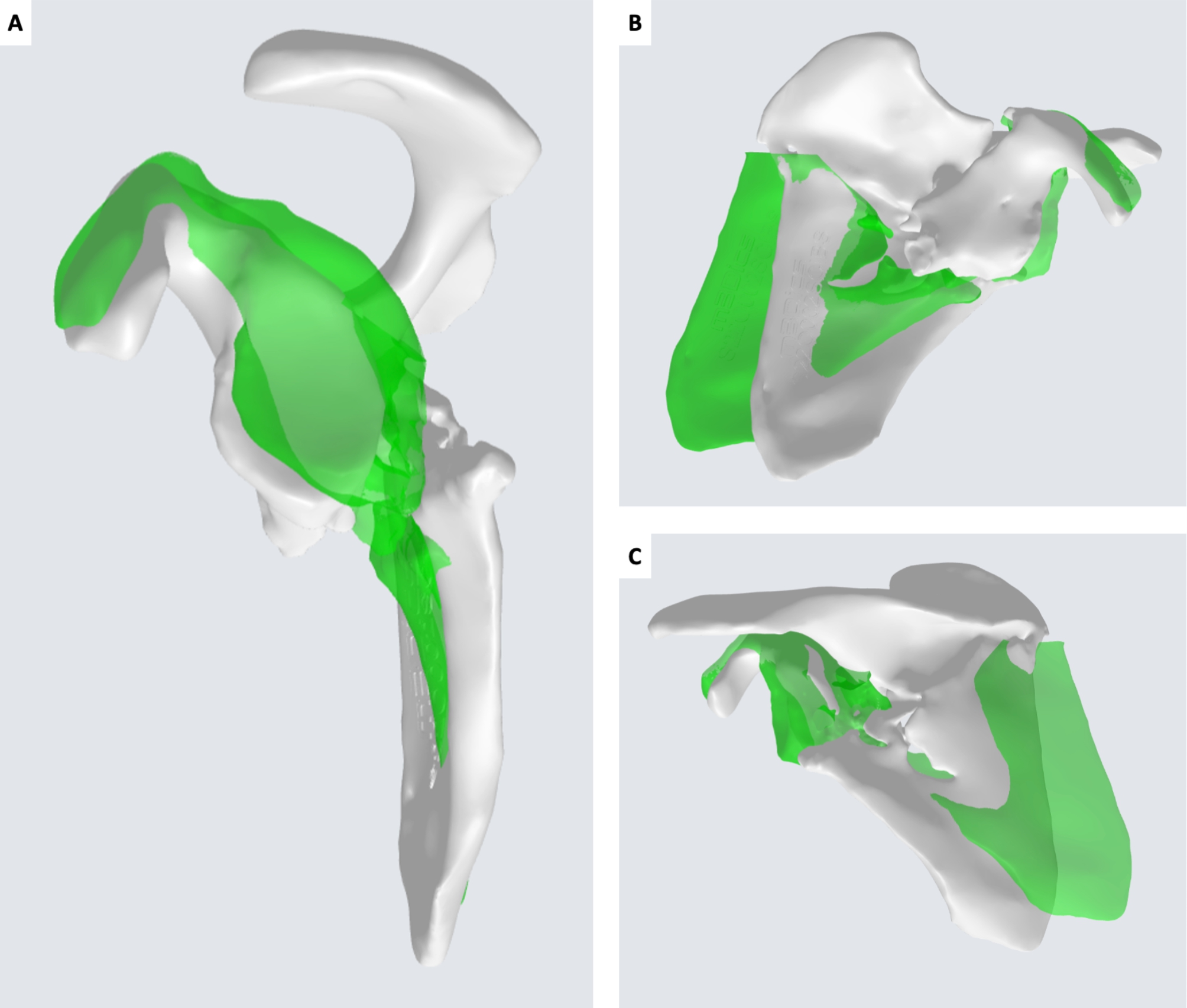
3D virtual rendering of the malunited left scapula (white) and mirroring with the healthy right scapula (green). A: sagittal view. B: coronal view from the front. C: coronal view from the back

CT DICOM files of both scapulae were sent to an external company (Medics Srl, Moncalieri, Italy) to develop a 3D model and an interactive virtual plan which allowed us to fully understand the deformity, by comparing the affected scapula to the healthy side in a mirroring procedure. The scapula deformity consisted in medialization and anterior displacement of the glenoid fragment, further enhanced by scapular body fragment lateralization. The scapula spine fragment showed forward inclination on the sagittal plane (Fig. 4).

**Fig. 5:**
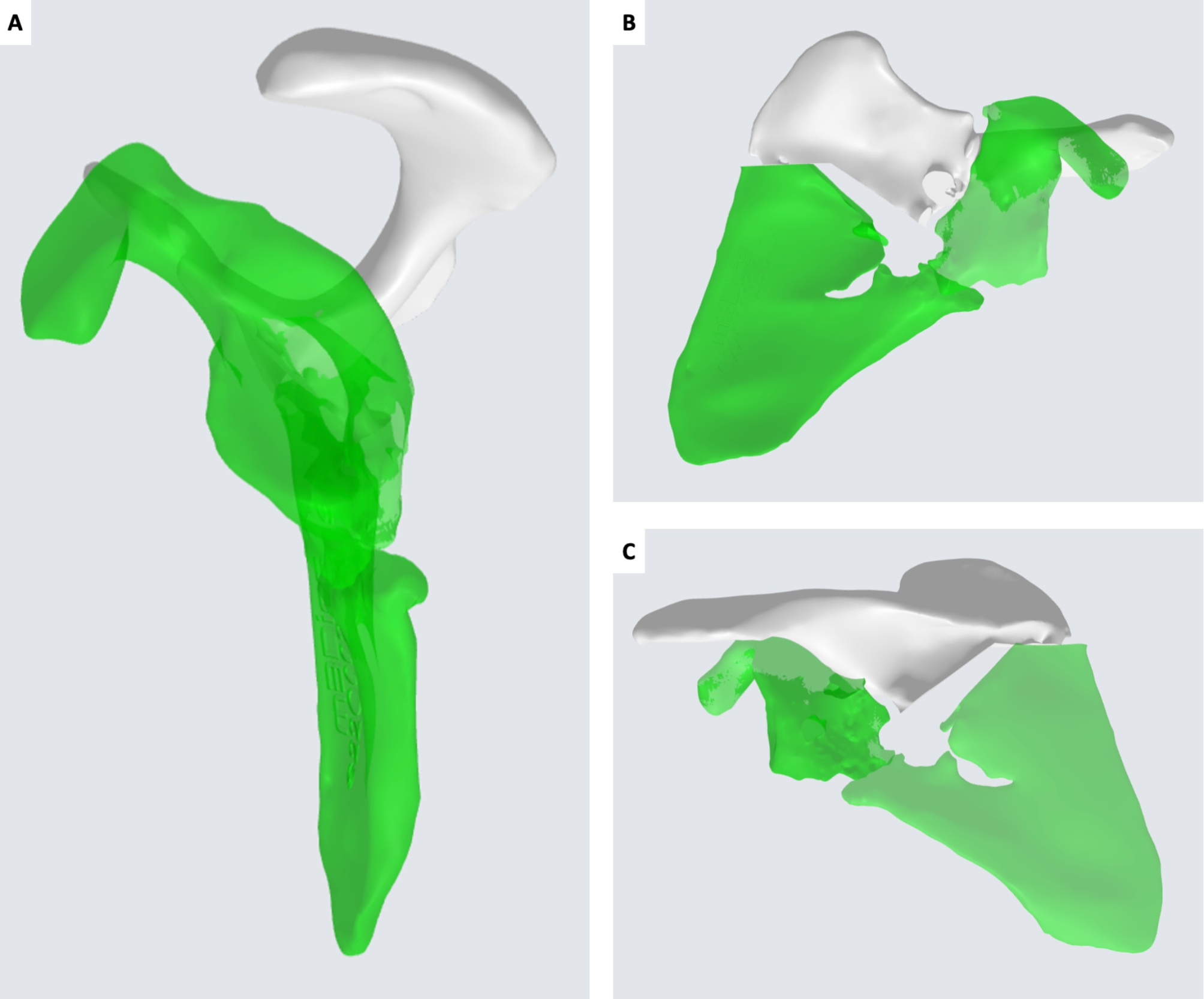
Definition of osteotomy lines and deformity correction. A: sagittal view. B: coronal view from the front. C: coronal view from the back

Surgical strategy consisted in separating the three main elements (glenoid, body and spine) through an osteotomy line between scapular spine and body and a second osteotomy between glenoid and scapular body (Fig. 5).

**Fig. 6:**
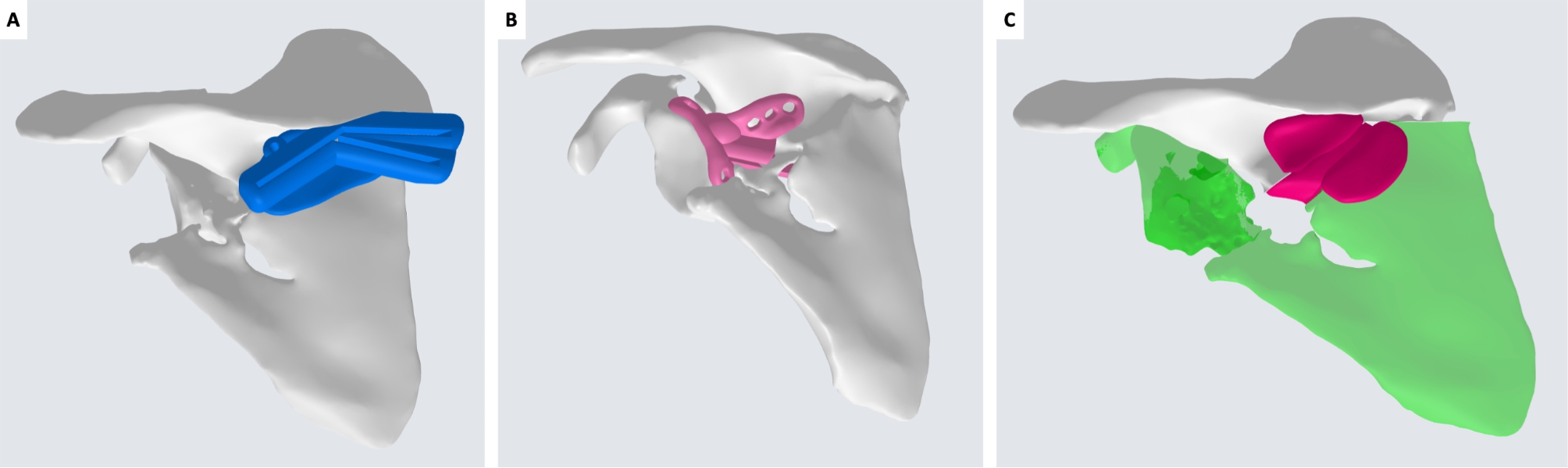
Patient-specific instrumentation to perform body-spine osteotomy and removal of a bone wedge (A), glenoid-spine osteotomy in “blind” anterior to the scapular spine fragment with protection of suprascapular neurovascular bundle (B), and patient-specific wedge spacer to hold the reduction during plate fixation (C)

Therefore, a first guide was created to separate scapular spine from scapular body along the previous fracture plane and to allow for lateralization correction through a bone wedge removal. The second guide, to perform an osteotomy between glenoid and spine segments, was adapted to the local complex morphology determined by callus formation and to the course of the suprascapular neurovascular bundle. A patient-specific wedge was created to assist holding the reduction during plate fixation (Fig. 6).

**Fig. 7:**
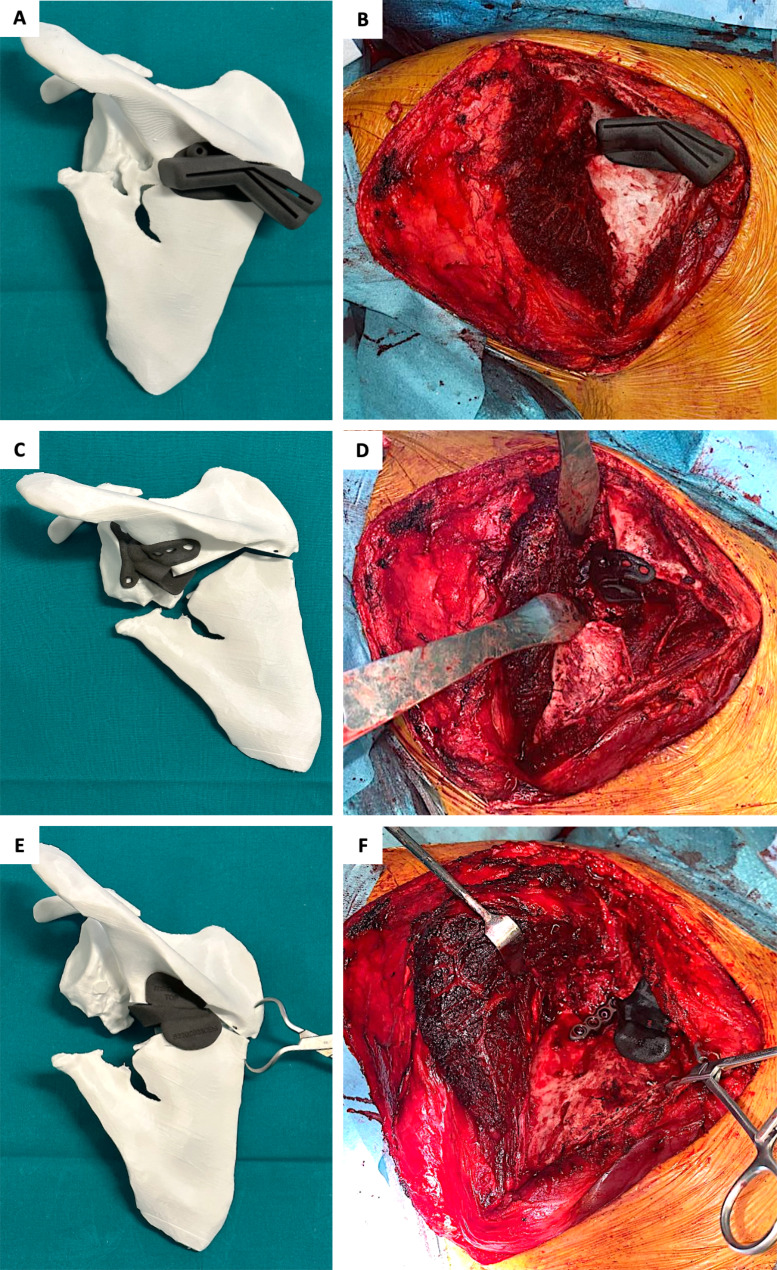
Positioning of the patient-specific cutting guides and wedge on the 3D model of the scapula and during surgery. A-B: first guide on the superomedial angle, allowing osteotomies and removal of a medial bone wedge. C-D: second guide directing the osteotomy between the glenoid and the spine segments. E-F: patient-specific wedge maintaining the angular correction with a lateral opening

Surgical procedure was performed under general anesthesia on a radiolucent table. The patient was positioned in prone decubitus and a traditional Judet approach to the scapula was performed through a boomerang-shaped incision. As planned with the interactive software and on the model, the first guide was positioned at the superomedial angle and fixed with a K-wire (Fig. 7A,B). Osteotomies between the scapular spine and the body were performed with an oscillating saw and an osseous wedge was removed. The second guide was positioned laterally, based on the lateral margin of the scapular spine and on the glenoid rim, and an osteotomy between the glenoid and the scapular spine segments was performed (Fig. 7C,D).

Once the three main segments were separated and mobilized, reduction sequence started at the vertebral border, with correction of coronal and sagittal deformity, using pointed clamps and a patient-specific wedge, created to hold the correction obtained (Fig. 7E,F).

**Fig. 8:**
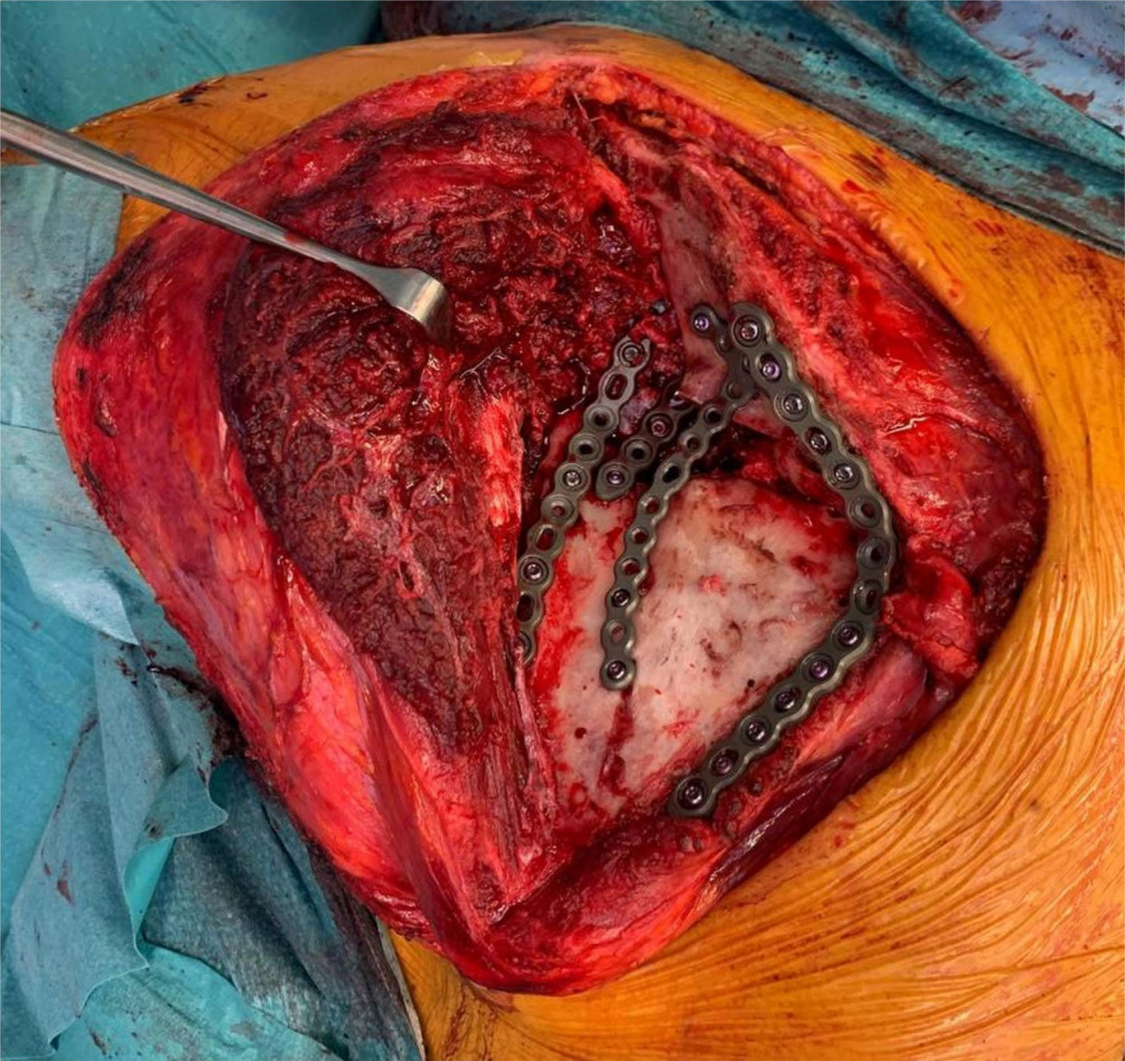
Intraoperative position of plates and screws after osteotomies and deformity correction

A 2.7-mm plate (Stryker VariAx 2 Mini Fragment Locking Plating System) was contoured around 90° to fit the angle between the vertebral border and the origin of the scapular spine and fixed with screws. Relationship and stability between the body and the spine segments was improved with a straight 2.7-mm plate (Stryker) contoured on the bony profile of the medial third of the scapular body and with a T-shaped 2.7-mm plate (Stryker) more laterally. Reduction between the obtained body-spine segment and the glenoid segment was performed with the aid of a Schanz screw implanted into the glenoid neck, with function of joystick, and a hook in the lateral border of the scapular body through a drilled hole. The Schanz screw allowed for direct manipulation of the glenoid segment, while the body was retracted distally and medially with the hook. Once a correct reduction was obtained, two 2.7-mm plates were contoured to fit the lateral border and fixed with screws (Fig. 8).

Scapular shape and hardware position were checked with fluoroscopy in Grashey, axillary and axillary Y views.

After copious irrigation with saline solution, posterior deltoid and infraspinatus were reinserted with #2 absorbable transosseous sutures through the spine and the vertebral scapular border. The wound was closed in layers. The upper limb was hold in a 15-degree abduction brace for 3 weeks. Passive and active rehabilitation were then started.

**Fig. 9:**
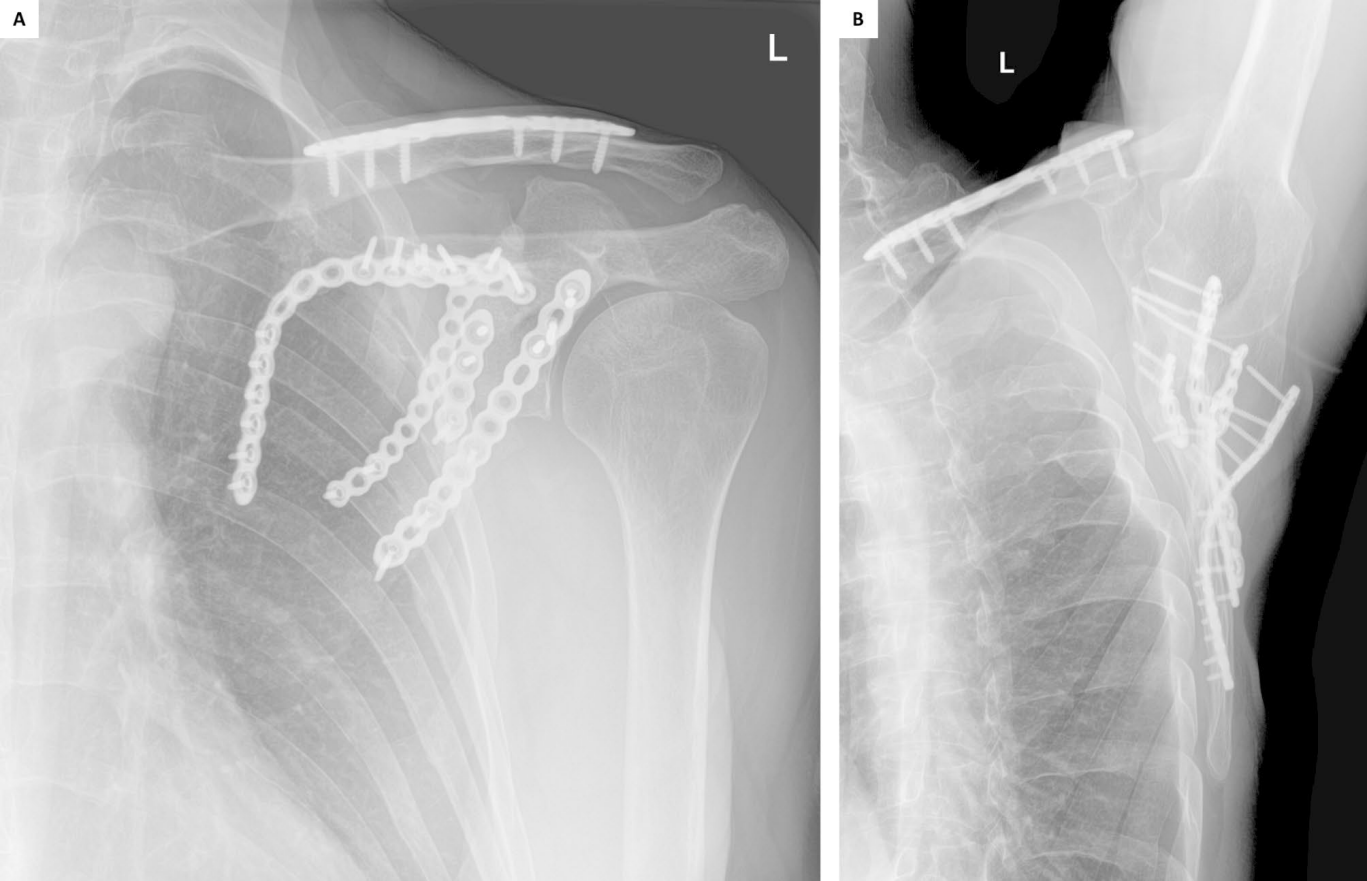
X-ray images of the left scapula 3 months after surgery. A: Grashey view. B: axillary Y view

Consolidation of the osteotomy was visible on x-rays after 3 months (Fig. 9).

**Fig. 10:**
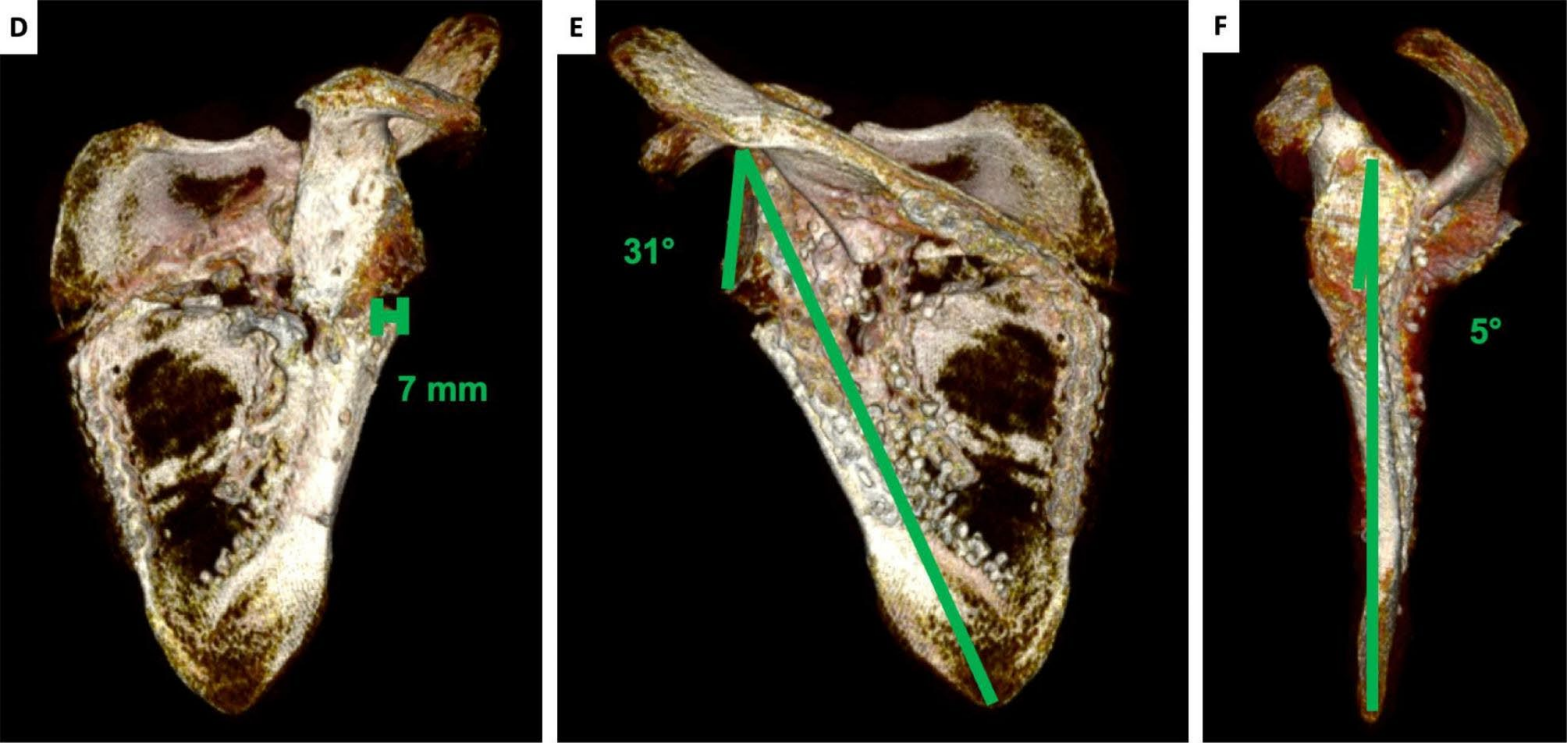
CT scan after surgery. A: lateral border offset 7 mm. B: glenopolar angle 31°. C: angulation deformity 5°

Clinical evaluation at 12-month follow-up showed an improved function of the shoulder girdle with 150° of active forward flexion, 30° of external rotation and internal rotation to the level of T12 (Fig. 10).

The DASH Score decreased to 28.3 and Total SPADI decreased to 21.5% (Total Pain Score 24%, Total Disability Score 20%).

**Fig. 11:**
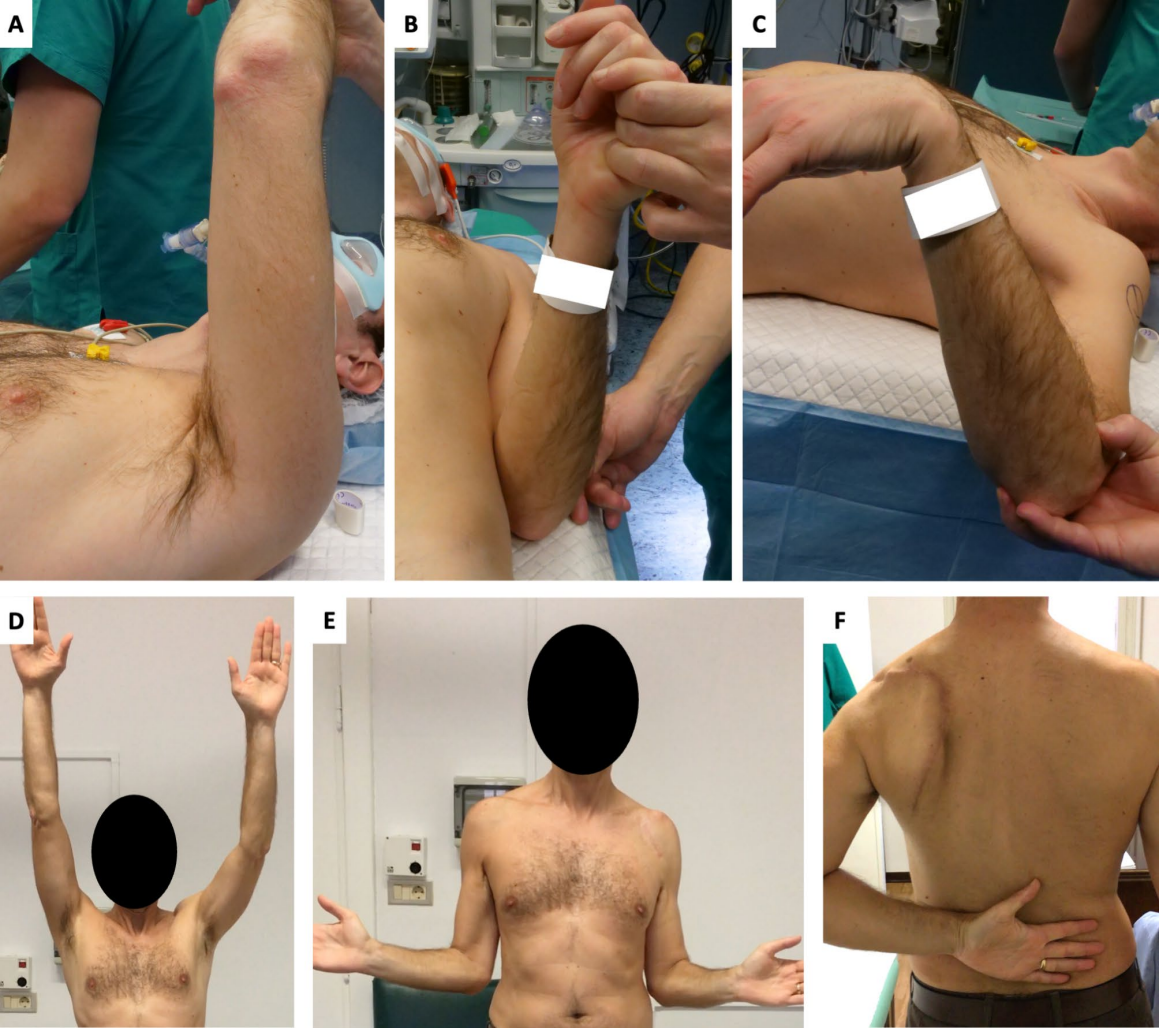
Clinical assessment of shoulder girdle function before surgery (A, B, C) and at 12-month follow-up (D, E, F). Forward flexion improved from 80° (A) to 150° (D). External rotation improved from 0° (B) to 30° (E). Internal rotation, initially limited to the buttock (C), reached the level of T12 (F)

Lateral border offset, glenopolar angle and scapular body angulation were recalculated on a postoperative CT: glenopolar angle increased to 31°, scapular body angulation was 5° and lateral border offset decreased from 53 to 7 mm (Fig. 11).

## Discussion and conclusions

Three-dimensional printing has an emerging role in preoperative planning and serves as an intraoperative support in crucial surgical steps. The use of 3D models in orthopedic surgery was shown to improve patient care and outcomes, decrease time spent in the operating room and enhance surgeon and patient education [23]. In the present case report, we showed the application of a 3D model to better understand post-traumatic scapula deformity in a malunion case. Mirroring procedure allowed to compare the affected side to the contralateral healthy side and to outline critical features of the malunion. Osteotomy lines and simulation of correction were planned on a virtual model and patient-specific guides were used to perform osteotomies and to assist correction maneuvers. This technology offered different advantages. First, malunion was analyzed in a 3D setting, which allowed to simultaneously understand the deformity and its correction on different planes. Furthermore, the creation of models was an opportunity for the surgeon to explain the procedure to the patient with a material support. Second, the virtual model was used as an interactive tool to simulate osteotomy lines, to accurately estimate angular and linear correction of the deformities, and to obtain a rendering of the expected result. Third, patient-specific cutting guides, designed according to the virtual model, allowed to precisely reproduce the planning and to perform osteotomies in a segment with such a complex anatomy like the scapula.

Indeed, some disadvantages of this approach should be considered. First, 3D models and guides have a cost; therefore, their use appears to be sustainable only for the treatment of complex and uncommon deformities. Furthermore, preparation of models and guides requires a time frame that makes this technology suitable for planned elective procedure, and less appropriate for urgent primary treatment of fractures.

Three-dimensional modelling and patient-specific guides are helpful tools to improve reliability of preoperative planning and surgery for the correction of scapular deformities. Largest series are needed to understand its application in the spectrum of scapula malunions and to develop reproducible strategies and treatment protocols.

## Data Availability

The datasets used in this study are available from the corresponding author on reasonable request.
